# Electrophysiological and Psychophysical Measures of Temporal Pitch Sensitivity in Normal-hearing Listeners

**DOI:** 10.1007/s10162-022-00879-7

**Published:** 2022-12-05

**Authors:** François Guérit, Andrew J. Harland, Matthew L. Richardson, Robin Gransier, John C. Middlebrooks, Jan Wouters, Robert P. Carlyon

**Affiliations:** 1 Cambridge Hearing Group, MRC Cognition & Brain Sciences Unit, University of Cambridge, Cambridge, England; 2 Department of Otolaryngology, University of California at Irvine, Irvine, CA USA; 3 Department of Neurobiology and Behavior, University of California at Irvine, Irvine, CA USA; 4 Department of Cognitive Sciences, University o f California at Irvine, Irvine, CA USA; 5 Department of Biomedical Engineering, University of California at Irvine, Irvine, CA USA; 6 Department of Neurosciences, ExpORL, Leuven, Belgium

**Keywords:** Emporal pitch perception, Psychophysics, Frequency following response, Auditory change complex

## Abstract

To obtain combined behavioural and electrophysiological measures of pitch perception, we presented harmonic complexes, bandpass filtered to contain only high-numbered harmonics, to normal-hearing listeners. These stimuli resemble bandlimited pulse trains and convey pitch using a purely temporal code. A core set of conditions consisted of six stimuli with baseline pulse rates of 94, 188 and 280 pps, filtered into a HIGH (3365–4755 Hz) or VHIGH (7800–10,800 Hz) region, alternating with a 36% higher pulse rate. Brainstem and cortical processing were measured using the frequency following response (FFR) and auditory change complex (ACC), respectively. Behavioural rate change difference limens (DLs) were measured by requiring participants to discriminate between a stimulus that changed rate twice (up-down or down-up) during its 750-ms presentation from a constant-rate pulse train. FFRs revealed robust brainstem phase locking whose amplitude decreased with increasing rate. Moderate-sized but reliable ACCs were obtained in response to changes in purely temporal pitch and, like the psychophysical DLs, did not depend consistently on the direction of rate change or on the pulse rate for baseline rates between 94 and 280 pps. ACCs were larger and DLs lower for stimuli in the HIGH than in the VHGH region. We argue that the ACC may be a useful surrogate for behavioural measures of rate discrimination, both for normal-hearing listeners and for cochlear-implant users. We also showed that rate DLs increased markedly when the baseline rate was reduced to 48 pps, and compared the behavioural and electrophysiological findings to recent cat data obtained with similar stimuli and methods.

## Introduction

Most periodic sounds that we encounter in everyday life contain both low-numbered harmonics, whose frequencies are resolved from each other by the peripheral auditory system, and high-numbered harmonics whose frequencies are unresolved. Decades of research have shown that pitch perception is dominated by the low-numbered resolved harmonics [1, 2], but that normal-hearing (NH) listeners can hear a musical pitch and discriminate changes in fundamental frequency (F0) even when only the unresolved harmonics are present [3–5]. The latter finding is of theoretical importance because it shows unequivocally that pitch can be conveyed using a purely temporal code, where no place-of-excitation cues are available. It is of practical value because the temporal code is responsible for the ability of cochlear implant (CI) users to perceive the pitch of broadband periodic sounds, such as speech. This is because CIs predominantly convey F0 using amplitude-modulated pulse trains [6, 7]. It may therefore be useful both to understand the neural basis of temporal pitch perception and to obtain objective measures in circumstances where behavioural responses are difficult to acquire, for example in young children or in research studies involving non-human participants. Here, we study temporal pitch processing in adult NH human listeners at three stages of the auditory system, from brainstem, to cortex, to perception, using very similar stimuli in each case, namely harmonic complexes that are bandpass filtered so as to contain only unresolved, high-numbered, harmonics.

Temporal pitch processing at the brainstem level was studied using the frequency following response (FFR). The FFR is a measure of the composite phase-locked response to sound and has a latency consistent with a dominant source in the upper brainstem, although there is evidence for one or more cortical components for frequencies below about 100 Hz [8–12]. Previous studies of the FFR to complex tones containing only unresolved harmonics have revealed some parallels with pitch perception of the same stimuli: for example, the effect of summing the harmonics in alternating vs. sine phase depends on frequency region and F0 in a similar way for the FFR and for pitch judgements [13]. As the FFR and pitch perception for these stimuli depend entirely on temporal processing, the comparison of FFRs to behavioural measures is arguably more straightforward than is the case for resolved harmonics, for which place-of-excitation cues may play a role [14].

To obtain a measure of temporal pitch perception at the level of the cortex, we measured the auditory change complex (ACC) to the same stimuli as those used to measure the FFR. The ACC reflects an evoked cortical response to a change in an ongoing sound and has been measured extensively both in NH and CI listeners. It has usually been recorded in response to manipulations that introduce changes in the cochlear excitation pattern—for example, a change in the frequency or level of a pure tone or of a complex tone containing resolved harmonics, or a change in the stimulating electrode of a CI [15–20]. Some experiments have revealed an ACC to changes in temporal properties of sound such as modulation rate or depth [21, 22], both in NH and CI listeners, but we are unaware of any study showing an ACC in NH listeners to stimuli similar to those used here and that employ rates that support temporal pitch perception. The present study is part of a project investigating whether such an ACC response can be obtained both in NH and CI listeners and used as a surrogate for psychophysical measures [23].

The ACC and FFR measures were compared to behavioural F0 difference limens (F0DLs) to a change in temporal pitch. The behavioural measures required listeners to detect a change in the F0 of an ongoing filtered pulse train. This paradigm differs from many (but not all) previous measures of the F0DL, which typically require listeners to compare the pitches of sequentially presented tones that are separated by silent intervals (e.g. [3, 24–26]. By using very similar stimuli for ﻿the ﻿FFR, ACC and perception, we could compare temporal pitch coding at multiple stages of the human auditory system and relate the results to those obtained in an analogous study in the cat [27], from which species we plan to obtain single-unit recordings. Our results demonstrate that an ACC can be observed to a change in temporal pitch and, by comparing the pattern of results to those observed for the FFR and in the psychophysical experiments, provide a potentially important link between physiology and perception. The development of these methods may also refine objective measures of temporal pitch perception in CI listeners, both by us [23] and others.

## Experiment 1: Simultaneous Recording of FFR and ACC in Response to Acoustic Pulse Trains

### Ethical Approval

The procedures for both experiments 1 and 2 were approved by the Cambridge Psychology Research Ethics Committee (project 2017.085), and written informed consent was collected prior to any testing.

### Methods

#### Participants

Thirteen listeners (8 females) aged 18–29 years (mean = 23) took part. Their audiometric thresholds were below 20 dB HL from 250 to 8000 Hz in the ear tested. They also underwent a short adaptive 2-interval 2-alternative forced-choice task to determine their pure tone detection thresholds (71% correct) at 10,800 Hz, corresponding to the upper edge of the passband of the highest bandpass-filtered stimulus used here (see below). The mean threshold at that frequency in the ear tested was 21-dB sound pressure level (SPL) with a standard deviation of 6.2 dB; all thresholds were below 30 dB SPL except for one participant whose threshold was 34 dB SPL.

#### Paradigm, Stimuli and Stimulating Equipment

**Fig. 1:**
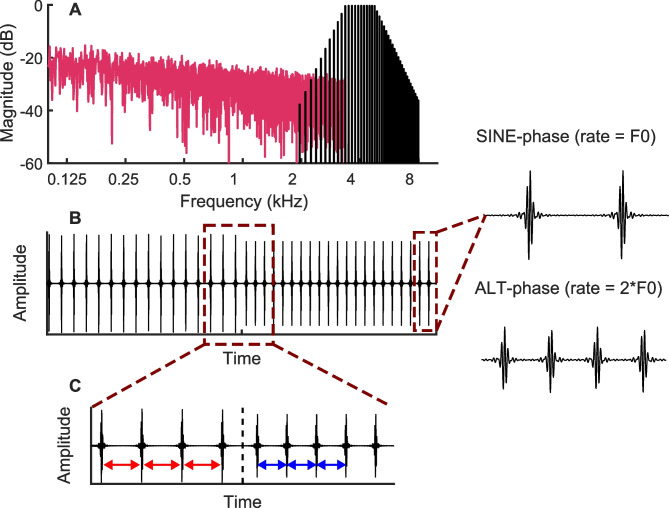
Schematic of the stimuli used in both experiments. Part **A** shows the spectrum of a pulse train filtered into the HIGH region plotted in black, with the pink noise plotted in pink. Parts **B** and **C** show the time waveform of the pulse train, without the pink noise, plotted on a coarse and on a fine time scale, respectively. The waveforms on the right of the plot illustrate pulse trains generated by summing harmonics in sine and in alternating phase

We used a continuous switching paradigm throughout experiment 1 (Fig. 1B). For each condition, the stimulus started with a pulse train of a given base rate and then switched rate every second (or two seconds in one condition) between that pulse train and a pulse train of the same root-mean-square (RMS) level but with a pulse rate that was approximately 36% or 66% higher. This allowed us to measure an ACC to each upward/downward change in pulse rate and also to measure the FFR in response to each segment of a steady-state pulse train.

**Fig. 2:**
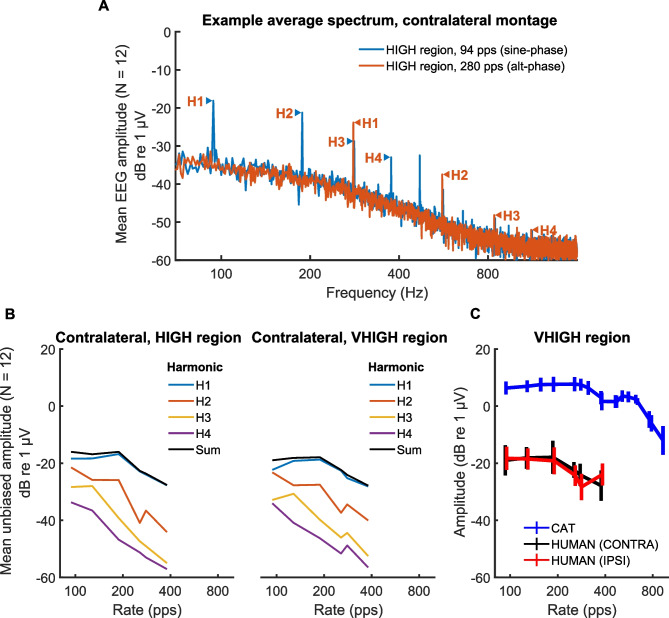
**A** Example average spectrum of the FFT for a 94-pps sine-phase pulse train (blue) and for a 280-pps alternating-phase pulse train (red) obtained with the contralateral montage. The pulse rate (H1) and harmonics 2–4 for each stimulus are indicated by labelled arrows. **B** Amplitude of the FFR component at the pulse rate (H1), harmonics 2–4, and the composite amplitude as a function of pulse rate. The left- and right-hand plots are for stimuli filtered into the HIGH and VHIGH regions respectively, both obtained with the contralateral montage. **C** Amplitude of the composite FFR peak as a function of pulse rate for the contralateral (black) and ipsilateral (red) montage. Data from the cat [27] are shown in blue

We generated the pulse trains by summing harmonics of a given F0 either in sine or in alternating phase (odd harmonics in the cosine phase, even harmonics in the sine phase). When summing the harmonics in the sine phase, the stimulus resembles a pulse train repeating at F0, while summing in alternating-phase results in a pulse rate having a rate equal to 2F0 (Fig. 1). Alternating-phase stimuli allow higher pulse rates to be presented while keeping harmonics unresolved [5]. Before summing, we adjusted the amplitude of the harmonics to create bandpass-filtered harmonic complexes (Fig. 1A). Amplitudes were constant between 3365–4755 Hz for the “HIGH” condition and 7800–10,800 Hz for the “VHIGH” condition, and decreased with a slope of 48 dB/octave beyond these cutoff frequencies. The frequencies for the HIGH region were chosen to be one octave below those used in our recent study with the cat [27], so as to roughly compensate for the different audibility ranges of the two species. The frequencies for the VHIGH region were the same as those used in the study by Macherey and Carlyon [28], which provides useful information on the resolvability of harmonics and on the limits of temporal pitch for these stimuli. Because each pulse train lasted exactly one second and all F0s were integer numbers, all pulse trains consisted of an integer number of pulses. We also ensured that each pulse train started and ended at the “silent” midpoint between two consecutive peaks in the waveform (cf. Fig. 1C). This eliminated the splatter that could otherwise occur at the transition point between pulse trains of different rates. Finally, each time the baseline and higher rates within a sequence was presented (i.e. every 2 s in most conditions) the polarity of the stimuli was inverted to reduce the strength of the stimulus artefact in the FFR measurements. We added pink noise to mask cochlear distortion products (Fig. 1A). To minimise masking of the stimulus itself, the pink noise was low-pass filtered at the same frequency as the lower cutoff of the bandpass filter used to generate the pulse trains in a given condition.

The main part of the experiment consisted of all possible combinations of three pulse rates (94, 188 and 280 pps) and two frequency regions (HIGH and VHIGH); they correspond to conditions 1 through 6 listed in the first six rows of Table 1. In these conditions, the pulse train switched every second between two rates that differed by 36%. Conditions 7 and 8 used a larger rate difference of 66% and with baseline rates of 188 and 280 pps filtered into the VHIGH region, and could be compared to conditions 5 and 6 which had the same base rates and frequency region but with the 36% rate change. Condition 9 was used to determine whether a larger ACC would be obtained with a slower switch rate, as has been used in some previous studies (e.g. [18, 22, 23]. We chose pulse rates so as not to be near (< 5 Hz) any harmonics of the 50-Hz domestic power supply in the UK. Pulse trains with rates below 188 Hz were generated in the sine phase, but for the faster rates, pulse trains had alternating phases with an F0 of half the desired pulse rate in order to prevent harmonics becoming resolved.

**Table 1:** List of conditions. Cutoff frequencies of the HIGH and VHIGH filter were respectively [3365–4755] Hz and [7800–10,800] Hz

Cond	Lower rate (pps)	Higher rate (pps)	Percent change	F0 – phase	Freq. region	Switch rate (Hz)
1	94	128	36	94 Hz – sine	HIGH	1
2	188	256	36	94 Hz – alt	HIGH	1
3	280	380	36	140 Hz – alt	HIGH	1
4	94	128	36	94 Hz – sine	VHIGH	1
5	188	256	36	94 Hz – alt	VHIGH	1
6	280	380	36	140 Hz – alt	VHIGH	1
7	188	312	66	94 Hz – alt	VHIGH	1
8	280	464	66	140 Hz – alt	VHIGH	1
9	94	128	36	94 Hz – sine	HIGH	0.5

Stimuli were presented in blocks, each of which included one presentation of each condition, in a random order. For all conditions except condition 9 each presentation consisted of 200 changes in pulse rate, so that there were 100 changes in each direction. For condition 9, which used a 2-s alternation, each presentation consisted of 100 changes (50 per direction). There was a 2-s silent gap between conditions within each block. Blocks were repeated 7 times (each time with a new random within-block stimulus order), so that, in total for each condition except condition 9, a given change direction was presented 700 times (100 times in each of seven blocks); for condition 9, each change direction was presented 350 times. Testing occurred over two sessions of 3 h, with the first session ending at the end of a block. This ensured that any change between the sessions (such as a slight difference in the positioning of the EEG cap) would affect all conditions equally.

We presented the stimuli monaurally (to the left ear for 6 participants and to the right ear for 7) over a shielded and grounded Etymotic ER2 earphone, connected to a Fireface UCX sound card and Tucker Davis Technologies HB7 headphone drivers. We calibrated the stimuli with a 2-cc earphone coupler so that the pulse trains alone (without the pink noise) had an overall (RMS) level of 60 dB SPL. The pink noise level was set so that the spectrum level (if it had not been low-pass filtered) at 4 kHz would be 13 dB SPL, i.e. 47 dB lower than the overall RMS of the pulse train (Fig. 1A; cf. [29]. Participants sat in a comfortable chair in an electrically-shielded, double-walled sound-attenuating booth. They watched a silent, subtitled movie or TV show for the duration of the experiment.

All recordings were obtained using an 8-electrode BioSemi Hyper Rate system [21, 30] sampling at 32 kHz with a 24-bit resolution. We placed pin electrodes via the cap at locations P9, P10, Iz, Cz, Fz and Fpz, and flat electrodes on the left and right mastoids. This ensured there would be two electrodes at each temporal bone (P9/P10 and the two flat electrodes) in case one of these electrodes became noisy during a recording session. All neural potentials—both for the FFR and ACC—are reported and analysed on a decibel scale, consistent with previous reports from our group [21, 23, 30–33]. This ensures that the size of the difference between two conditions is not affected by factors that influence the overall gain between the neural generator and neural response, such as between-subject differences in the distance or orientation of neural generators relative to the recording electrodes.

#### FFR Analysis

For the FFR analysis, we kept the recordings at their original sampling rate (32 kHz). No filtering apart from the 6500-Hz anti-aliasing filter was applied. Traces of all channels were referenced to electrode Cz, and segmented into 1-s epochs, starting at each rate change. We subtracted the DC component from the whole epoch and averaged together all epochs with the same rate for each condition (half of them being of opposite polarity by construction). We obtained the amplitudes and phases at the pulse-rate frequency and the 2nd-4th harmonics from the corresponding bins of a fast Fourier transform (FFT) of that averaged epoch (cf. Fig. 2). We compared the linear power at each of these frequencies to that of the adjacent 6 bins (3 on each side, each 1-Hz wide) using an *F* test. An *F* ratio greater than 10.92 (*p* < 0.01, approx. 10 dB of unbiased signal-to-noise ratio) was deemed significant [34]. We computed a composite value of the FFR by summing the unbiased power (linear power at the signal bin minus the average linear power at the neighbouring bins) at the base rate and harmonics 2, 3 and 4. Note that in some cases (mostly at harmonics 3 and 4), the power at the signal bin was smaller than at the neighbouring bins. In that case, the unbiased linear power for that harmonic was set to zero. Finally, phase values at the pulse-rate frequency from all conditions common to both filter regions were calculated and unwrapped to compute the group delay separately for each frequency region. All calculations of group delay were corrected by 1 ms to compensate for the lag between the electrical input to an ER2 phone and the stimulus reaching the end of the plastic tubing, as observed by Elberling et al. [35].

#### ACC Analysis

For the ACC, we down-sampled the recordings to 2 kHz using the BioSemi Decimator utility. We averaged data from channels P9, P10 and Iz in the time domain, and referenced these data to channel Cz. We then bandpass filtered the data between 1 and 16 Hz and segmented them into epochs starting 200 ms before and ending 500 ms after each rate change. The 10% of epochs with the largest peak-to-peak amplitudes (for a given condition and direction of rate change) were removed. Finally, we averaged the resulting epochs together into one waveform per condition/rate change direction.

The amplitude of the ACC was determined by measuring the RMS value within the 50–250-ms window after the change in rate. This was then compared to a baseline estimate calculated over the window spanning 200 ms prior to each rate change (cf. [36]. This was preferred over peak picking N1 and P2 values because of the overall low amplitudes measured and so as to avoid any effects of choosing any particular method of identifying peaks and troughs.

### Results

#### FFR

We observed a robust FFR across the tested range of 47–380 pps, with the amplitude declining somewhat at the highest pulse rates. We focus on the results from the contralateral montage (i.e., contralateral mastoid vs Cz) since it yields good FFR/ACC responses and is commonly used in CI EEG experiments, including our own recent research, so as to minimise electrical artefacts (e.g. [23, 37]. Broadly similar results were obtained using the ipsilateral montage; the effect of montage on FFR latency and amplitude are discussed briefly below.

Figure 2A shows the FFT of the FFR in two example conditions, namely 94-pps and 280-pps pulse trains filtered into the HIGH region. The labelled arrows indicate the presence, for both stimuli, of peaks corresponding to components at the pulse rate (H1) and at the 2nd-4th harmonics (H2–H4). It can be seen that for each stimulus the component amplitude decreases with increasing frequency. Note also that for the 280-pps stimulus, for which the components were summed in alternating phase, there is no visible component at the 140-Hz F0, consistent with previous measures of the FFR with unresolved components [13, 32]

Figure 2B shows the FFR amplitude (contralateral montage) obtained at each pulse rate and frequency region for the first six conditions shown in Table 1. The different-coloured lines show the amplitude of the FFT at the pulse rate and at each of harmonics 2, 3 and 4, with the sum of these values shown in black. We plot the amplitude as a function of pulse rate rather than F0 because, for unresolved harmonics, the FFR is expected to be driven by the pulse rate, which for alternating-phase stimuli is equal to 2F0, as illustrated in Fig. 2A. The FFR is largest at the pulse rate (blue lines) but is usually above the noise floor even at the higher harmonics, reflecting the fact that the FFR waveform is not purely sinusoidal. We use the composite FFR in all the following analyses and figures. The black and red lines in Fig. 2C show the effect of montage on the variation in FFR with pulse rate; the blue lines show data from the cat [27] and will be considered in the “Discussion” section

**Fig. 3:**
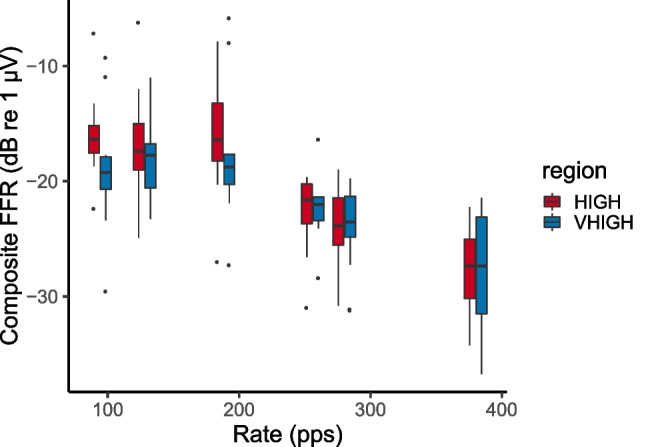
Composite FFR as a function of stimulus pulse rate. Data from the HIGH and VHIGH regions are shown in red and teal bars respectively. For this and all other box-and-whisker plots in this article, the solid horizontal lines show the median, and each box extends from the median to plus-and-minus the inter-quartile range (IQR)

Figure 3 shows the composite FFR amplitude for conditions 1–6. A two-way (rate X region) repeated-measures ANOVA revealed a significant effect of rate (*F*(5, 55) = 30.7, *p* < 0.001) This was expected based on previous research [38–40], and the overall decrease with increasing rate likely reflects low-pass filtering by the head [12] and possibly a decrease in phase locking and/or synchrony with increasing pulse rate. In addition, as shown by Tichko and Skoe [12], the FFR can show marked local fluctuations in amplitude—likely reflecting the interaction between multiple neural generators—when measured using pure tones with a wide range of closely spaced frequencies, and it is possible that some of the particular rates used here corresponded to a local peak or trough. The main effect of frequency region was not significant (*F*(1,11) = 3.47, *p* = 0.09). There was a significant interaction between rate and region, reflecting the slightly steeper decline in FFR with increasing rate for the HIGH compared to the VHIGH region (*F*(5,55) = 2.53, *p* = 0.04).

**Fig. 4:**
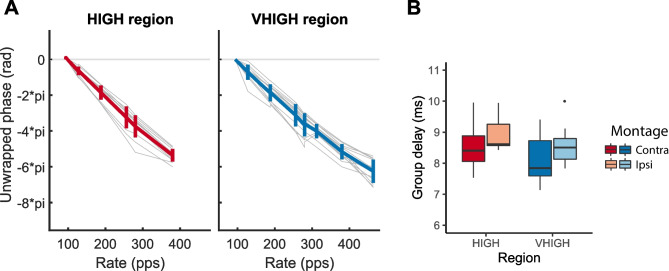
**A** Unwrapped phase-vs-frequency plots for the FFRs obtained with the contralateral montage and in the HIGH and VHIGH regions. **B** Group delays derived from the phase plots in the HIGH and VHIGH region and for the contralateral and ipsilateral montages

Finally, we computed the group delay in each frequency region and for two electrode montages, namely P9 and P10 each referenced to Cz. These were defined as the contralateral and ipsilateral montages, or vice versa, depending on which ear was being stimulated. The unwrapped phase plots for the contralateral montage are shown in Fig. 4A. The group delays are shown in Fig. 4B and were analysed using a two-way repeated-measures ANOVA with two factors: filter region and EEG electrode montage. The group delay was significantly shorter for the VHIGH than for the HIGH frequency region (*F*(1,11) = 24.8, *p* < 0.001). This is expected from cochlear mechanics (given that basilar-membrane filters are broader at higher frequencies [41, 42] and because the group delay of a filter decreases with increases in its bandwidth. The effect of montage just failed to reach significance (*F*(1,11) = 4.38, *p* = 0.06).

#### ACC

**Fig. 5:**
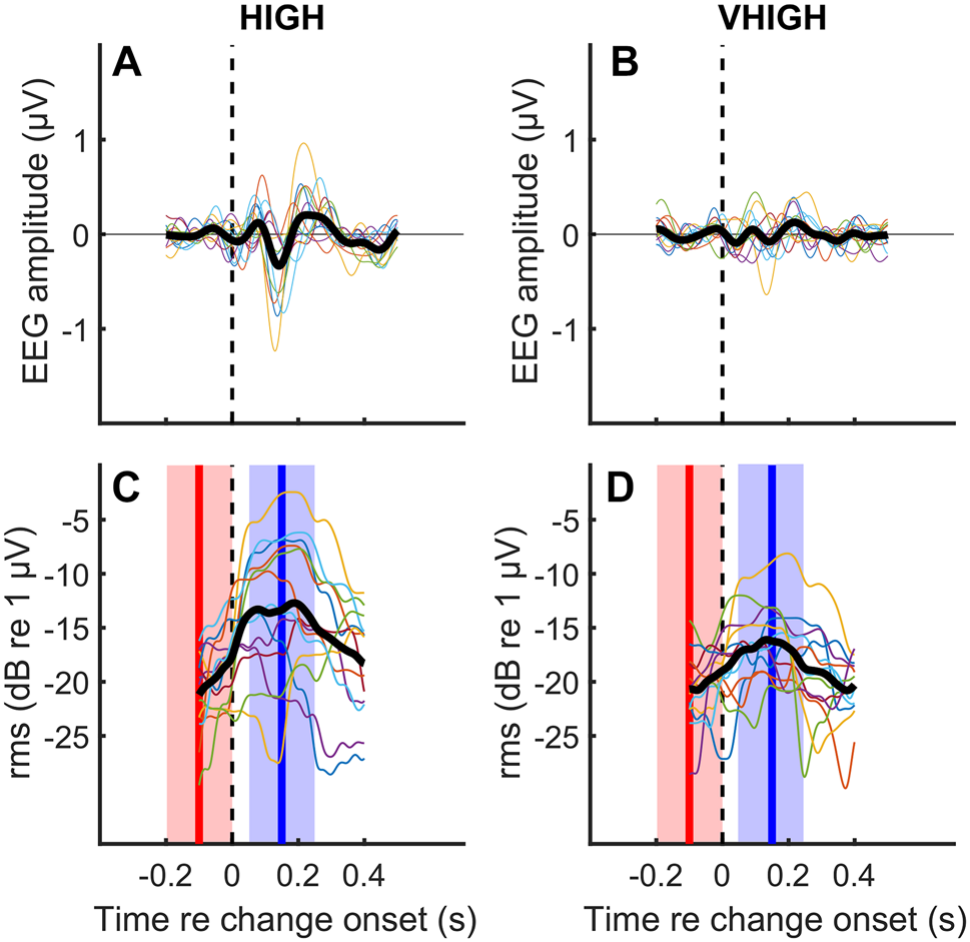
Parts **A** and **B** show the ACC averaged across all pulse rates and for increasing and decreasing rate changes for conditions 1–6 of experiment 1 and for the HIGH and VHIGH regions respectively. Individual data are shown by the faint coloured lines and mean data are shown by the thick black lines. Parts **C** and **D** are analogous to parts **A** and **B** but show the RMS calculated over a 200-ms running window, and with the baseline period and window of interest shown by the red and blue shaded areas, respectively

**Fig. 6:**
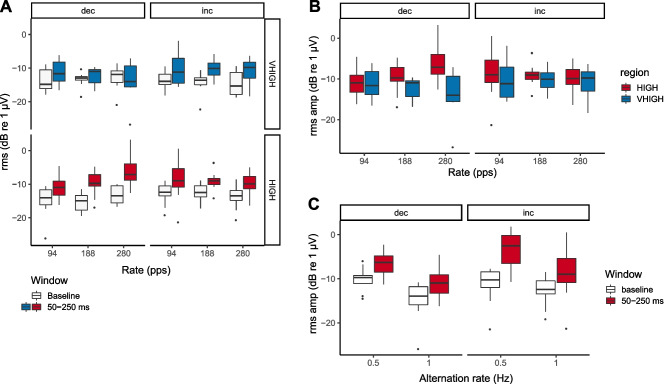
**A** The RMS ACCs measured during the window of interest are shown as a function of pulse rate, separately for rate decreases and increases, in the HIGH and VHIGH regions by the solid red and teal boxes, respectively. The RMS values measured during the baseline period are shown by the white boxes. **B** ACCs from the region of interest re-plotted from part **A** so as to aid a visual comparison between HIGH and VHIGH regions. Data are shown for conditions 1 to 6 for which the rate change was 36%. **C** RMS ACCs in target (solid bars) and baseline (open bars) intervals for conditions 1 and 9, which differed only in switch rate

The thick black lines in Fig. 5 show the grand average response across listeners for the HIGH (panel A) and VHIGH (panel B) filter region, averaged across base rates and direction of rate change. Data from individual participants are shown by the fainter coloured lines. Although there is evidence for the typical ACC morphology (negative and positive peak about 100 ms and 200 ms following the rate change) for individual listeners, the average excursions are fairly small (< 1 μV peak-to-peak) and the morphology in the average data is less clear, due partly to differences in latency between participants. We therefore quantified the ACC in terms of its RMS (in dB re 1 μV) within 50 to 250 ms after the onset, rather than extracting the peak values, for which the latencies were sometimes hard to define. The evolution of the RMS value is illustrated in panels C and D, which show the RMS calculated over a running 200-ms window, and where it can be seen that the RMS increases after the pulse-rate change (dashed vertical line). To check for the presence/absence of an ACC in our recordings, we tested whether the measured RMS was significantly larger in the window of interest (50–250 ms, blue shaded areas in Fig. 5C, D) than obtained from the baseline period (200-ms window prior to the onset, red-shaded areas), where no ACC is expected. Figure 6A compares the RMS in the baseline window (open bars) versus the window of interest (blue and red bars). A 4-way repeated-measures ANOVA (window location × frequency region × rate × change direction) revealed a highly significant main effect of window location (*F*(1, 12) = 36, *p* < 0.001). This confirms the presence of an ACC to a change in pulse rate. We therefore performed all subsequent analyses on the scores from the window of interest only. We also performed an analysis on the N1–P2 amplitude difference, with the N1 and P2 defined as the minimum and maximum amplitudes in the periods 50–150 ms and 150–250 ms after the rate switch, respectively, and with “control” measures obtained over periods 200–100 ms and 100-ms before the switch. Although we restrict our discussion to the RMS measures, the results of the N1–P2 analysis were broadly similar, including a highly significantly larger amplitude after than before the switch.

The left- and right-hand panels of Fig. 6B shows the ACC as a function of pulse rate and frequency region for decreases and increases in rate, respectively. These data are re-plotted from the solid boxes in Fig. 6A to aid comparison between the HIGH and VHIGH results. A repeated-measures ANOVA on the RMS values (in dB) showed a significant 3-way interaction (rate × region × direction, *F*(2,24) = 5.73, *p* = 0.009) and a significant effect of region (*F*(1, 12) = 8.0, *p* = 0.015)—reflecting larger ACCs in the HIGH than in the VHIGH region—but no other significant effects. The 3-way interaction appears to be driven by a large response to the decrease in pulse rate in the HIGH region at 280 pps. A possible reason for this large response is that, at 280 pps in the HIGH region, harmonics are more likely to be partially resolved than at lower rates or for stimuli filtered into the VHIGH region. This might cause the number of harmonics interacting within an auditory filter on the low-frequency slope of the excitation pattern to transition from 3 for the baseline rate to 2 for the higher rate. Because the components were summed in alternating phase, this could cause the beating rate to halve [28]. However, this does not explain why the large response was observed only for the decreasing rate switch, and not for the increasing rate switch.

The solid bars in Fig. 6C show the ACCs for conditions 1 and 9, which differ only in the switching rate. The mean ACC in condition 9, which had a 0.5-Hz switching rate, was − 5.2 dB re 1 μV, significantly larger than the value of − 9.9 dB in condition 1, which had a 1-Hz switch rate. This difference was confirmed by a 2-way (direction × switch rate repeated-measures ANOVA, which revealed a main effect of switch rate (*F*(1,12) = 48.5, *p* < 0.001). A separate 1-way ANOVA performed on the 0.5-Hz switch-rate data from condition 9 showed a significantly larger ACC for increases than for decreases in pulse rate (*F*(1,12) = 14.3, *p* < 0.01). However, this finding should be treated with caution because the 2-way ANOVA described above did not reveal a significant interaction between direction and switch rate (*F*(1,12) = 0.25, *p* = 0.63). The higher overall amplitude for the slower switch rate came at the expense of collecting only half as much data in the available time, thereby increasing the measurement noise. To evaluate this further we calculated the signal-to-noise ratio (SNR) as the dB difference between the target and baseline intervals (spanning 50 to 250 ms and − 200 to 0 ms re the rate change) for each participant and switch direction and for conditions 1 and 9, which differ only in switch rate. A 2-way repeated-measures ANOVA on switch rate and direction revealed no main effect of switch rate (*F*(1,12) = 1.5, *p* = 0.25) on the SNR. The effect of switch direction just missed significance (*F*(1,12) = 4.0, *p* = 0.07) and there was no significant interaction (*F*(1,12) = 3.2, *p* = 0.1).

**Fig. 7:**
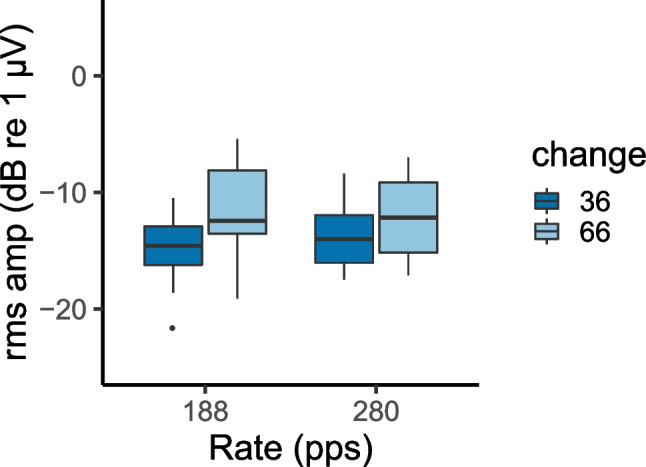
ACC RMS amplitude for 36% and 66% rate changes and for 188- and 280-pps baseline rates. Stimuli were filtered into the VHIGH region

Figure 7 compares the ACC to 36% and 66% changes for 188- and 280-pps pulse trains filtered into the VHIGH region corresponding to conditions 5–8 in Table 1, pooled across upward and downward pulse-rate shifts. A repeated-measures ANOVA showed that the ACC was significantly greater for the larger rate change (*F*(1,12) = 11.4, *p* = 0.005), visible in the difference between the dark- and pale-blue box plots. There was no significant effect of rate nor an interaction between the two factors. The results are consistent with a more salient change leading to a larger ACC.

## Experiment 2: Psychophysical Measure of Rate Change Difference Limens

### Rationale

The aim of experiment 2 was to provide psychophysical measures of temporal pitch encoding that used similar stimuli and presentation methods to the electrophysiological measures from experiment 1. As such, it was important to use a task in which listeners detect a change in the pulse rate of an ongoing stimulus, rather than to compare the pitches of two sounds separated in time, which is the method used in many F0-discrimination studies (e.g. [3, 5, 43]. This latter “sequential comparison” approach differs from the ACC paradigm in two potentially important ways: (i) it often, but not always, uses a 2-interval forced-choice design which requires the listener to encode the direction of the pitch change, whereas the ACC only requires that the presence of a change is detected; (ii) there is evidence that the auditory processing of temporal pitch is “sluggish”, and this will likely impair the detection of a change in temporal pitch in an ongoing pulse train more than for a sequential comparison task [44, 45]. A further consideration arose from our initial attempts to measure performance in a sequential comparison 2-interval forced-choice task, where subsequent analysis of the results revealed contextual effects of the previous trial on the response to the present trial. Specifically, we found that when roving the overall pulse rate from trial to trial, an increase in pulse rate relative to the previous trial resulted in a tendency to judge the second sound in the trial as higher in pitch, with the opposite bias following a decrease in pitch from the previous trial. A similar context effect for pure tone stimuli was observed for a subset of participants by Matthias and colleagues [46] and for all 14 participants tested in a slightly different paradigm by Arzounian et al. [47]. Such context effects could complicate the comparison between behavioural and electrophysiological measures of pitch coding.

### Methods

**Fig. 8:**
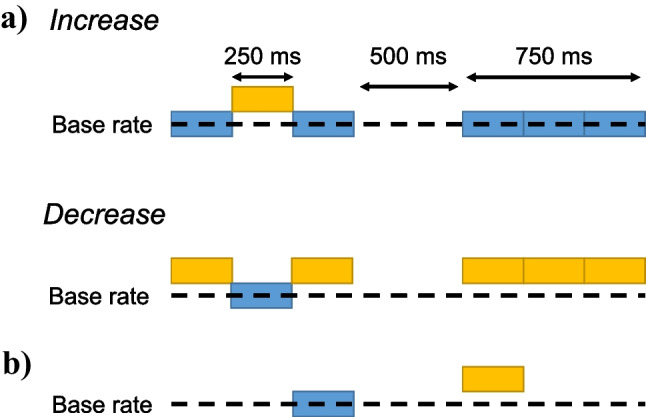
Schematic of the trial structure in the change detection (**A**) and the sequential comparison (**B**) conditions of experiment 2

The main part of the experiment used the task shown in Fig. 8A. Participants clicked on a virtual button on a computer screen so as to identify the interval in which the pulse rate changed from its baseline value to a different value and back again, with a total stimulus duration of 750 ms, and with the pulse-rate changes occurring after 250 and 500 ms. These durations were adjusted for each stimulus to the nearest integer number of pulses in each segment. In each trial, the “baseline rate” was defined as the lower rate present in the trial (blue in Fig. 8A). The trial started at that baseline rate for the increasing rate change, or at a higher rate (yellow in Fig. 8A) for the decreasing rate change. This meant that, for a given rate difference, the two rates to be discriminated were the same for an increasing- and for a decreasing rate change (Fig. 8A). The location of the interval (first or second) containing the change was randomised from trial to trial and correct-answer feedback was provided after each trial. This change detection task is broadly analogous to the ACC, although to save time and minimise boredom a shorter stimulus duration was used. Because participants were only required to detect a change in one interval but not to identify its direction, we expected this to reduce context effects related to the relationship between the direction of between vs within-trial changes [46, 47]. Furthermore, within a trial, the pulse rate at the end of each stimulus and at the start of the next was always the same, thereby eliminating the possibility that the direction of change between the two halves of the trial influenced the detection of possible rate changes during the second stimulus. The absence of sequential context effects was indeed confirmed by a trial-by-trial analysis of results described in a later section.

Except for the shorter duration and faster switch rate, the method of stimulus generation was identical to that used in conditions 1–6 of experiment 1. As in that experiment, stimuli were presented monaurally via an Etymotic ER2 earphone and in a background of low-pass-filtered continuous pink noise. The level and filtering of the noise were the same as in experiment 1. For both frequency regions, we added an extra condition where stimuli were summed in sine phase and with a pulse rate of 47 pps. This was inspired by our findings in the cat [27] that both the ACC and psychophysical performance (d’) were reduced at the lowest rate tested (94 pps in cat) compared to higher pulse rates; we wished to see whether we could observe a similar deterioration for human listeners by including an even lower pulse rate, consistent with experiments on the lower limit of temporal pitch [48, 49]. Hence, the main part of the experiment had eight conditions, consisting of all combinations of four baseline pulse rates (47, 94, 188, 280 pps) and two frequency regions (HIGH vs VHIGH).

The rate differences within a trial were 2.5, 5, 10, 20 or 40% of the baseline rate. Stimuli were presented in blocks of 80 trials, with each block consisting of 4 baseline rates × 2 change directions × 5 rate differences × 2 signal interval positions in random order. The frequency region was fixed within a block and switched every 7 blocks, with testing starting with the HIGH region for half the participants and with the VHIGH region for the other half. Blocks were repeated until participants had completed an average of 126 trials (ranging from 112 to 140 trials) for each combination of frequency region, baseline rate and rate difference and for a total of 28 or 35 blocks depending on the participant. Ten normal-hearing participants took part, eight of whom were recruited from a volunteer panel, had not participated in experiment 1, and were reimbursed for their time. Participants P5 and P6 were authors AH and FG respectively; they were experienced in psychophysical tasks including an initial experiment (not described here) involving similar stimuli and procedures. Participants performed only a small amount of practice before data collection began; however, as noted below, there were no measurable practice effects during this (main) part of the experiment. Sigmoidal fits were applied to the data for each combination of participant, frequency region and baseline rate, and difference limens were obtained via interpolation of the 75% correct point. If performance did not reach 75% at the largest (40%) rate difference tested, then the DL was estimated by extrapolation of the sigmoidal fit.

At the end of the main part of the experiment, two additional sets of measures were obtained with 94-pps sine-phase stimuli filtered into the HIGH frequency region. First, six participants repeated the change detection task and performed a sequential discrimination task in which each trial contained two 250-ms pulse trains separated by a silent gap of 500 ms (Fig. 8B). Note that in both of these tasks only a single baseline rate was tested, unlike the main part of the experiment where the baseline rate varied from trial to trial. The baseline and signal rates were the same as in the change detection task and the participant was required to indicate which stimulus had the higher pitch. Second, five of these participants also repeated the change detection task again, along with a version of the task in which the duration of each 250-ms stimulus segment was tripled, so that the duration of each stimulus was 2.25 s.

### Results

**Fig. 9:**
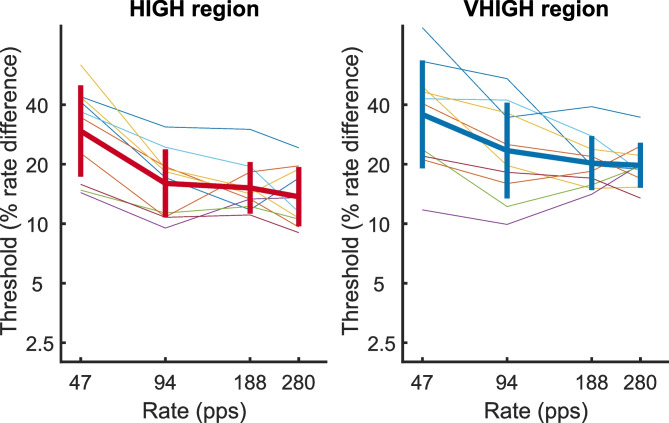
Rate DLs for the main part of experiment 2 as a function of baseline pulse rate and for the HIGH and VHIGH regions in the left- and right-hand plots respectively. Data for individual participants are shown by the faint coloured lines while mean data are shown by the thick lines. Error bars in Figs. 9, 10 and 11 show plus and minus one standard deviation

**Fig. 10:**
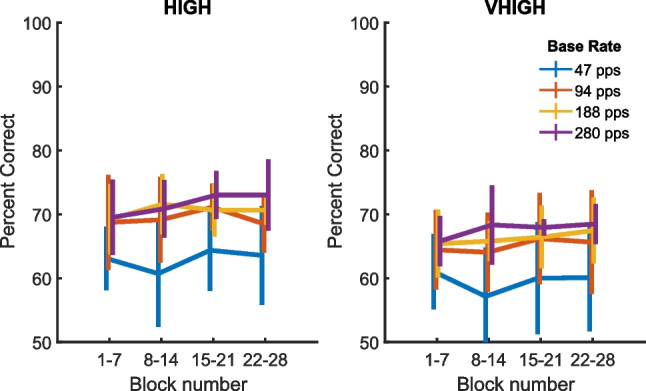
Percent-correct performance in the main (change detection) part of experiment 2 as a function of block number. The left- and right-hand panels show data for stimuli filtered into the HIGH and VHIGH regions respectively. Baseline pulse rates are indicated by the colours of the lines. All data were averaged over percentage rate differences and both directions of rate change

The rate DLs for the main part of the experiment, collapsed across the direction of change, are shown in the left- and right-hand parts of Fig. 9 for the HIGH and VHIGH region respectively. In a minority of cases (10% in the HIGH region and 20% in the VHIGH region), the psychometric function for a given participant did not reach the 75% correct value at which we defined the DL, and in these cases, the DL was obtained by extrapolation. There is considerable variability in the overall level of performance across participants (faint lines), and the mean rate DLs (thick lines) are considerably higher than the values of approximately 5% obtained in many sequential discrimination tasks for complex tones consisting of unresolved harmonics and with pulse rates in the range between about 60 and 300 pps [5, 26, 44, 50]. Nevertheless, the variation in DL with baseline rate and frequency region was sufficiently consistent within each listener for a 2-way repeated-measures ANOVA to reveal highly significant main effects of rate (*F*(2,27) = 13.0, *p* = 0.001) and of frequency region (*F*(1,9) = 31.2, *p* < 0.001); the interaction was not significant (*F*(3,27) = 1.0, *p* = 0.4) (note that all DLs in experiment 2 were converted to logarithms prior to analysis). Bonferroni-corrected pairwise comparisons for the effect of pulse rate revealed that the DLs for the 47-pps rate were significantly higher than for all other rates (*p* < 0.001, = 0.010, = 0.014 re rates of 94, 188 and 280 pps, respectively) and that DLs for no other rates differed significantly from each other. Hence the main effect of rate was driven by the higher DLs at 47 pps. Further evidence for the consistency of the data comes from across-subject correlations in the rate DLs between the HIGH and VHIGH regions. To test this, we performed a univariate ANOVA with the log of the DL in HIGH region as dependent variable, log of the VHIGH-region DL as covariate, participant as a random factor, and rate as a fixed factor. This allowed us to assess the significance of the covariate using an *F* test (*F*(1,19) = 39.2, *p* < 0.001) and to derive the correlation of 0.82 using the sum-squared errors for the covariate and error term [51]. To test for the presence of practice effects, we averaged the data over blocks 1–7, 8–14, 15–21 and 22–28, and plotted the data as a function of these 4 periods and for each baseline pulse rate in Fig. 10, separately for the HIGH and VHIGH regions. It can be seen that there is no marked change in performance over time. A 3-way repeated-measures ANOVA revealed significant effects of frequency region and of pulse rate, but no significant effect of time period (frequency region: *F*(1,8) = 19.9, *p* < 0.001); rate: *F*(3,24) = 10.6, *p* < 0.001; time period: *F*(3,24) = 2.6, *p* = 0.08).

**Fig. 11:**
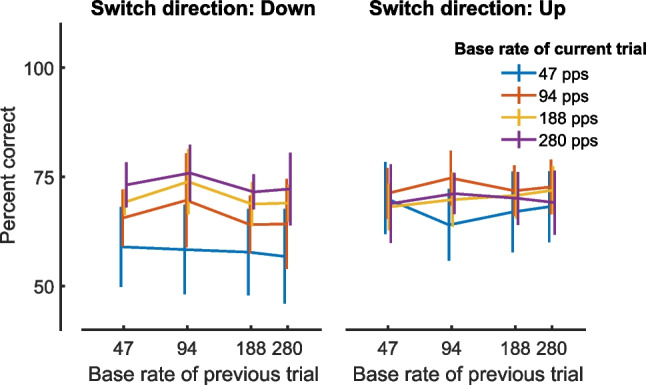
Performance on experiment 2 as a function of the baseline rate on the previous trial, with baseline rate on the present trial indicated by the colour of each line. The left- and right-hand plots show data for trials in which the first switch in the signal interval was downward or upward, respectively. Data are averaged over all different percent rate differences and are shown only for stimuli filtered into the HIGH region

Figure 11 shows the percent-correct scores from the main part of the experiment collapsed across rate changes separately for each baseline pulse rate, as a function of the baseline pulse rate on the previous trial. These data are shown for the HIGH frequency region for trials with increasing and decreasing pulse rates, respectively. The results for the VHIGH region were very similar and are not plotted. A context effect similar to that reported for sequential discrimination tasks would manifest as better performance for rate decreases when the previous trial had a high rate, and with the opposite being true for the detection of rate increases. It can be seen that the rate used in the previous trial did not affect performance, thereby confirming that our method successfully avoided context effects related to congruence of between- and within-trial frequency changes.

To determine whether the direction of the rate change influenced performance, we performed a univariate ANOVA on the psychometric functions (percent-correct scores) with frequency region, baseline rate, rate difference and direction as fixed factors and participant as a random factor. An advantage of this approach, compared to analysing DLs, is that it does not depend on extrapolation of the psychometric functions to obtain DLs when performance is poor. Similar to the DL analyses the univariate ANOVA revealed highly significant effects of baseline rate (*F*(3,27) = 12.8, *p* < 0.001) and of frequency region (*F*(1,9) = 21.9, *p* = 0.001), reflecting the poorer performance at 47 pps compared to higher rates and in the VHIGH compared to the HIGH region, and no significant rate × region interaction. The direction of rate change did not produce a significant main effect (*F*(1,9) = 2.7, *p* = 0.153) but did interact both with frequency region (*F*(1,9) = 12.7, *p* = 0.006) and baseline rate (*F*(3,27) = 17.5, *p* < 0.001). The interaction between change direction and frequency region reflected the fact that performance was better for upward than for downward changes by 3.4% in the HIGH region with a smaller difference of 0.8% in the VHIGH region. The interaction between change direction and rate reflects the fact that performance was better overall for increasing rate changes at 47 and 94 pps, with the opposite tending to be the case at 280 pps. However, the effect of direction at any one rate was very small, having a maximum value of about 5%. The effect of change direction also interacted significantly with participant, indicating the presence of idiosyncratic but reliable differences. The only other significant main effects and interactions involved the size of the rate change. We consider these effects to be trivial, as performance was expected to increase with larger rate changes and because other effects are unlikely to be present for the smallest rate changes where performance was close to chance.

**Fig. 12:**
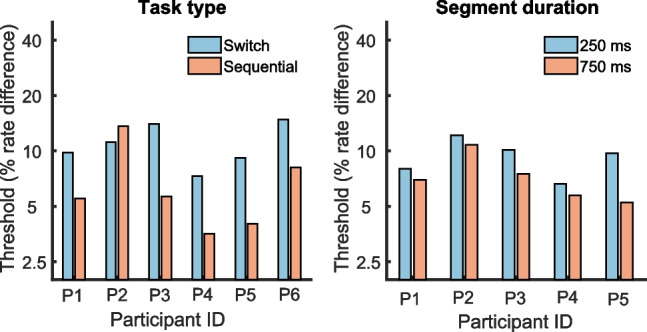
Results of the additional parts of experiment 2 showing the effects of task type (left-hand panel) and the duration of each segment of the stimulus (right-hand panel) for each participant

The left-hand panel of Fig. 12 shows the rate DLs for the additional experiment that compared performance for the 94-pps HIGH-region condition in the change detection and sequential comparison tasks. A two-tailed paired-sample *t*-test revealed that DLs were significantly lower in the sequential-detection task, where the geometric mean DL was 6.1% compared to 10.7% for change detection (*t*(5) = 3.52, *p* = 0.02). In addition, the change detection DLs were significantly lower than obtained for the same participants in the main part of the experiment (geometric mean = 15.9%, compared to 22.7% in the main experiment, paired-sample 2-tail *t*-test *t*(5) = 5.95, *p* < 0.002). Given the absence of practice effects during the main part of the experiment, we argue in the “Discussion” section that this was probably due to the use of a single baseline rate in the supplementary experiment compared to the use of multiple baseline rates within each block of the main experiment. There was a strong across-participant correlation between change detection DLs in the main part of the experiment and those obtained in the supplementary comparison between the change detection and sequential comparison tasks (*r* = 0.90, *p* < 0.02). DLs were lowest for participants P5 and P6; this was also the case for the main part of the experiment. This could be due to their extensive training in this and/or other psychophysical tasks, greater motivation (as they were also the experimenters), and/or to innate differences or experience (for example P6 is an amateur classical musician). Finally, the right-hand panel of Fig. 12 shows that, for the change detection task, increasing the duration of each segment from 250 to 750 ms produced a small but significant reduction in the DL, from a geometric mean of 9.1 to 7.0% for the 5 participants tested (paired-sample 2-tailed *t*(4) = 2.82, *p* < 0.05). To summarise, (i) rate DLs for change detection dropped when participants were re-tested in blocks of trials where the same baseline rate and frequency region were used for every trial, (ii) the variation in performance across participants remained very similar between the two testing sessions, (iii) switching to a sequential discrimination paradigm further reduced rate DLs to an average of 6.1%, only slightly higher than the typical values of 4–5% previously reported for similar stimuli and using an adaptive procedure, and (iv) a modest improvement was reported for the change detection task when the segment duration was increased from 250 to 750 ms. The results suggest that any benefit of not having to identify the direction of frequency change in the change detection task is outweighed by an effect of temporal sluggishness.

## Discussion

The present study obtained measures of temporal pitch perception at three levels of processing, namely the brainstem, cortex and perception. We start by comparing the data obtained with each measure to previous findings in the literature, including results obtained not only with NH humans but also with CI users and from our recent study with cats [27]. We end with a discussion of the relationship between the different measures.

### Comparison with Previous Results

#### FFR

Our measures of the FFR in response to unresolved complex tones produced results broadly consistent with previous measures obtained with normal-hearing human listeners. Frequency components were summed in alternating phase for pulse rates of 188 pps and higher, and with the F0 equal to half the pulse rate, so as to ensure that the harmonics were unresolved by the auditory system. As illustrated in Fig. 2A, the FFT to the FFR for these stimuli consisted of peaks at the pulse rate and its multiples, with no peak at the F0. This is consistent with the results of Krishnan and Plack [13], who presented filtered harmonics and orthogonally manipulated the F0, frequency region and phase, and who found that the lowest spectral component in the FFR to alternating-phase complexes corresponded to the F0 for resolved complexes and to the pulse rate for unresolved complexes. A similar finding was reported by Gockel et al. [32], who filtered 75-dB SPL alternating-phase harmonic complexes between 3900–5400 Hz. The amplitude of the FFR component corresponding to the pulse rate for the start of a 213-pps pulse train was about − 17 dB re 1 μV in the Gockel et al. study. This is similar to that observed here for a 60-dB SPL 188-pps 1-s pulse train, although about 5 dB higher than that observed for a 256-pps pulse train (Fig. 2). Although comparisons between studies will be affected by differences in recording methods and montages, the overall amplitude of the FFRs reported here appear broadly similar to those reported previously despite the long signal duration and moderate stimulus level used.

Our results are also broadly consistent with FFR measures obtained from two different populations. We recently measured the FFR, ACC and behavioural discrimination in normal-hearing cats, using stimuli that were similar to those in our HIGH frequency region but with the bandpass-filter and pink noise filters shifted up by one octave so as to account for the differences in auditory frequency range between the two species [27]. As with the present data, the cat FFR to alternating-phase complexes showed frequency components at multiples of the pulse rate (rather than the F0) and decreased in amplitude with increasing frequency. Measurement of the group delay yielded an estimate of 5.3 ms, shorter than the shortest value of 7.6 ms observed here, which was obtained with the contralateral montage and for stimuli presented in the VHIGH region. The decrease in FFR amplitude with increasing rate above about 200 pps observed here differs from that in the cat study, where FFRs were larger overall and remained constant in amplitude up to about 600 pps, as shown by the blue line in Fig. 2C. The between-species difference may have at least partly been due to the smaller head size of the cat.

The methods developed here formed the basis for our measurements of the electric FFR (eFFR) and eACC for presentation of single-electrode pulse trains to human CI listeners [23]. The highest rate at which Gransier et al. could obtain a reliable eFFR was limited to 168 pps due to the pulse-blanking procedure used to eliminate the electrical artefact. Estimation of the group delay from the 128- and 168-pps trains tested yielded a value of 5.7 ms, with the 94-pps data showing a phase lag consistent with a longer, cortical, latency. This differs from the shortest group delay of 7.6 ms observed here, which occurred for the contralateral montage and stimuli filtered in the VHIGH region, and which was constant between 94 and 464 pps (Fig. 4). The fact that this delay was observed for rates up to 464 pps makes it unlikely that it was affected by a cortical source, for which evidence has only been reported for frequencies below about 100 Hz [8, 11, 12]. Rather, the longer group delay compared to the 5.7 ms for CI listeners is likely at least partly due to the travelling wave delay, which is absent for CI listeners and which may also account for the slightly longer group delay observed here in the HIGH compared to the VHIGH region. For example, Elberling et al. [41] estimated travelling-wave delay from narrowband ABRs recorded by Don et al. [52], and obtained values of 2.5 and 1.7 ms at frequencies corresponding to the geometric centres of our HIGH and VHIGH regions respectively, although somewhat shorter estimates were obtained using data from other methods that they used.

#### ACC

The ACC has previously been observed using both EEG and MEG in response to a change in a wide range of stimulus parameters, including the introduction of a silent gap, changes in stimulus level, a switch from one speech sound to another, and shifts in the frequency of pure tones and in the F0 of complex sounds [19, 53]. In most of these cases, the change that elicits the ACC will also have produced a change in the level or shape of the peripheral excitation pattern. In other words, the firing rate of one or more auditory nerve fibres will change, and this change in the excitation pattern will have been conveyed progressively through the multiple tonotopically organised stages of the auditory system. This is true even for changes in the F0 of complex sounds and even when those sounds are bandpass filtered, as long as some low-numbered resolved harmonics are present [54]. Another measure of cortical processing, the mismatch negativity (MMN), has been shown to be sensitive to differences in purely temporal pitch [55], but differs from the ACC in the need for the participant to build up an internal template from a series of many “standard” stimuli to which a rare deviant stimulus can be compared. It was therefore not obvious that one could observe an ACC to a change in a purely temporal pitch, under conditions that do not produce a change in the peripheral firing rate profile. Our finding of an ACC to changes in temporal pitch is however broadly consistent with two related strands of evidence. The first strand is the observation of ACCs to changes in the temporal envelope of sounds, albeit at envelope repetition rates that are too low to elicit a pitch. For example, Undurraga et al. [22] measured an ACC to a change in modulation rate from 20 to 35 Hz (and vice versa) imposed on a 500-Hz sinusoid. Other researchers have used EEG and reported ACCs to the onset of AM imposed on a stimulus, although this inevitably introduces a momentary increase or decrease in level it has been argued that the ACC reflects the response to the AM per se rather than to this transient change [21, 56]. The second strand comes from MEG studies showing a cortical response to the transition between a white noise and an iteratively rippled noise (IRN) or between two IRNs that elicit different pitches [57–59]. IRNs can be produced by delaying and adding a white noise to itself and repeating this process multiple times (iterations); this leads to a sound with a pitch roughly equal to the reciprocal of the delay, and a pitch strength that increases as more iterations are added [60]. However, although the pitches of IRNs are consistent with temporal models of pitch perception, it should be noted that as more iterations are added the stimulus increasingly resembles a random-phase harmonic complex tone and that pitch is strongest when frequencies corresponding to low-numbered harmonics of the perceived pitch (1/delay) are present. MEG data on the change response to IRNs have not usually bandpass filtered the stimuli at sufficiently high frequencies to remove harmonics below about the 10th and that are potentially resolved by the peripheral auditory system, and we are not aware of any such study that also used a background noise to mask any low-numbered harmonics that are introduced by the cochlear nonlinearity. Hence, it is not possible to conclude unequivocally that change responses to IRNs reflect changes in purely temporal pitch rather than changes in harmonics that are resolved by the peripheral auditory system.

Our results showed that it is indeed possible to record a consistent and reliable ACC to a change in purely temporal pitch (i.e. for changes in the rate of unresolved harmonics). The size of the ACC—with an RMS typically of about 0.3 μV (− 10 dB re 1 μV) in the HIGH region—is smaller than has often been observed to stimuli leading to salient perceptual changes, and that also produce changes in the peripheral excitation pattern [15–20]. One reason for this may be that the change in the pulse rate of an unresolved complex tone is not very perceptually salient; indeed, experiment 2 showed that the detection of such changes in the rate of a pulse train is quite poor, even when participants were experienced in the task and where the standard stimulus was the same within each block (Fig. 12, left-hand panel). In addition, the salience of the pitch change may have been reduced by the low sensation level and noise background needed to ensure the absence of detectable cochlear distortion products. The peak-to-peak values of the ACCs to a change in modulation rate from 20 to 35 Hz in the Undurraga et al. [22] study were approximately 3–4 μV for a 100% modulation depth, where the modulation rate change would have been highly salient, but was only about 0.6 μV for a 50% modulation depth. Another reason likely arises from our paradigm in which the pulse rates switched every 1 s, compared to the 2 s used by, e.g. Undurraga et al. [22],our comparison of conditions 1 and 9 in experiment 1 showed that doubling the switch time increased the amplitude of the ACC, albeit at the expense of halving the number of ACCs so that there was no significant improvement in SNR.

One final possibility to be considered is that the ACC observed here may have resulted from changes in loudness between different pulse rates, rather than from a change in pitch. For example, increases in F0 will lead to slightly fewer harmonics interacting on average within each auditory filter, leading to small reductions in the “peakiness” of the auditory filter output, and some loudness differences have been previously observed between stimuli having the same RMS level but differing greatly in crest factor [61]. Evidence against this loudness explanation comes from the finding that increases in level result in larger ACCs than decreases in level, a finding originally obtained in NH humans [17] and subsequently replicated in CI humans [62] and in NH cats [20, 27]. No such asymmetry was observed for our pulse-rate changes in the main part of experiment 2, although a significant difference in this direction was obtained in condition 9, which used 94-pps high-region stimuli presented with a 0.5-Hz switch rate. It is also worth noting that the absence of a direction effect in our main data also differ from our findings in the cat using similar stimuli, and where we observed markedly larger ACCs for upward than for downward frequency shifts [27].

#### Psychophysics

The psychophysical DLs obtained in the main part of experiment 2 were considerably higher than the values of around 5% typically obtained for unresolved harmonics using a sequential comparison paradigm and for pulse rates above about 90 Hz [5, 26, 50]. They did, however, agree qualitatively with previous evidence [48, 49] showing that DLs increase substantially at lower pulse rates, as evidenced here by the significantly higher DLs at 47 pps compared to all higher rates. The results are also qualitatively consistent with the increase in DL with increasing frequency region for 75-pps pulse trains reported by Deeks et al. [29], although the overall DLs were higher in the present study. One reason for the higher overall DLs reported here is revealed by the fact that for the 94-pps HIGH-region stimulus, DLs were significantly greater for our change detection task than for the more traditional sequential discrimination paradigm (Fig. 12, left-hand panel). A relevant finding is that DLs for the detection of F0 modulation for unresolved complexes, which is another measure of the processing of ongoing pulse-rate changes, are also substantially higher than for sequential comparisons between steady stimuli; this effect is much larger for complexes containing only unresolved harmonics than for those containing resolved harmonics [44]. However, Plack and Carlyon [44] attributed this finding largely to the fact that, for sinusoidal FM, the stimulus spends only a short amount of time near the peaks of the frequency excursion, and reported similarly poor performance, specific to unresolved harmonics, for a sequential comparison task in which the stimulus duration was reduced from 200 to 50 ms. Note that in our change detection paradigm, there were three 250-ms steady-state portions of the signal, which in principle would provide ample time for the auditory system to extract the temporal pitch in each segment [44]. Hence, the difference in performance between the change detection and sequential comparison tasks is likely caused primarily by participants being unable to “extract” each 250-ms segment and calculate its pitch, rather than in the pulse rate not remaining constant for long enough for participants to integrate pitch information. However, tripling segment duration from 250 to 750 ms did produce a modest reduction in the DL, from about 9% to about 7% (Fig. 12, right-hand panel), suggesting that having a longer stimulus over which to integrate information likely had an additional effect.

We found no significant improvement in DLs as a function of block number during the main part of experiment 2, suggesting that the high DLs reported were not simply due to an unfamiliarity with the stimuli or with the requirements of the task or procedure. Our finding that performance on the change detection task improved when participants were re-tested during a later session (Fig. 12, left-hand panel) is reminiscent of the “delayed gains” that can be observed on some auditory tasks many hours after training has ceased [63]. However, these gains have usually been reported after extensive training with a single stimulus, whereas the baseline rate varied substantially from trial to trial in the main part of experiment 2. We believe that a more likely explanation for the difference observed on re-testing is that, in this later session, only a single baseline rate was tested within a single block, and that the often substantial between-trial changes in rate impaired performance.

The practice of mixing F0s between blocks in the main part of experiment 2 is qualitatively similar to that of roving overall frequency from trial to trial, a manipulation that has been extensively investigated using pure tone stimuli (e.g. [46, 64, 65]. Research by Mathias and colleagues suggests that there are at least two ways in which roving may impact performance [46, 66]. One of these, which appears to be specific to tasks that require the listener to identify the direction of a frequency change, is that performance is better when frequency changes in the same direction between and within trials. It is most common in subsets of participants—originally identified by Semal and Demany [67]—who are overall worse at identifying the direction of frequency changes than in detecting the presence of a change. Even though this effect occurs for continuous as well as for discrete frequency changes [46], our analysis found no evidence that it occurred in the change detection task of experiment 2 (Fig. 11). A second effect of roving can impair performance even for experienced listeners who have no particular difficulty in identifying the direction of frequency change and even in tasks that do not require the change direction to be identified [46, 65]. We believe that it is this second effect—which may arise from the ability to develop and store an accurate representation of frequency in fixed-stimulus paradigms—that is responsible for the reduction in F0DLs in the mixed-block stage of experiment 2 and the single-F0 supplementary stage of that experiment.

Our psychophysical data also parallel those obtained recently in the cat using analogous stimuli. Richardson et al. [27] used a change detection task and found that sensitivity (d’) to 36% and 66% rate changes was significantly lower for a 94-pps baseline pulse rate compared to higher rates. Our listeners’ DLs were roughly similar at 94 pps compare to higher rates, but we observed a marked increase as the pulse rate was reduced further to 47 pps. This is consistent with the idea that there is a central lower limit to temporal pitch [48, 49] and that this limit is lower in humans than in cats, perhaps due to the different range of F0s to which each species is exposed, e.g. via conspecific vocalisations. It is also worth noting that, broadly consistent with the cat psychophysics, the cat ACC also decreased at low pulse rates, leading to the prediction that, had we measured the human ACC at 47 pps, its amplitude would also have been reduced compared to that at higher baseline rates.

### Comparison Between Measures

The behavioural and ACC data were broadly consistent with each other in showing larger ACCs and lower DLs in the HIGH than in the VHIGH region, and with no effect of baseline rate over the range (94–280 pps) common to the two experiments.

A possible difference between the FFR and our other two measures is suggested by the significant effect of frequency region for the ACC and behaviour and which was absent for the FFR. However, the fact that one effect reaches statistical significance while another fails to does not mean that the two effects differ significantly from each other. We therefore performed an additional analysis to determine whether the effect of frequency region was indeed significantly greater for the ACC than for the FFR. Because the two measures yielded different dependent variables, we converted the difference (in dB) between the HIGH and VHIGH regions into an effect size, for each participant and measure, by dividing it by the standard deviation of all participants’ scores in both regions. A two-way (measure × rate) repeated-measures ANOVA revealed that the effect of measure was not significant (*F*(1,11) = 2.3, *p* = 0.16), and so we have no evidence that the FFR and ACC depended differently on frequency region. There was no significant effect of rate (*F*(2,22) = 1.3, *p* = 0.29) and the rate × measure interaction just failed to reach significance (*F*(2,22) = 3.5, *p* = 0.05).

The most marked difference between the FFR data on the one hand and the ACC and behaviour on the other is the large effect of pulse rate only for the FFR. Tichko and Skoe [12] measured the FFR for pure tones for a wide range of closely spaced frequencies and observed local variations in amplitude, which they modelled as interactions between multiple generators, combined with an overall low pass characteristic that they attributed to the capacitive properties of the skull. Although the ACC will also be affected by the low-pass filtering properties of the skull, this will not cause the ACC to vary with frequency as it is a change response whose frequency content does not vary with pulse rate. Filtering by the skull will also not of course affect behavioural FDLs. FFRs might also be affected by the synchrony of firing either between neurons in the same neural location (e.g. inferior colliculus) or between different neural generators (e.g. [12, 32], and these factors will not necessarily affect the ACC or perception. Note, however, that the rate-dependence of the FFR does not preclude its use for examining interventions that might influence phase locking at the brainstem level and that might have knock-on effects on the representation of pitch at higher levels of auditory processing. For example, changes in the FFR as a result of pharmaceutical treatment, auditory deprivation or novel methods of stimulating the auditory nerve (e.g. [68–72] may be interpreted as a change in sub-cortical processing, provided that the pulse rates or frequencies used are sufficiently high to render a cortical contribution unlikely. Combining FFR and ACC measures may then allow a simple and non-invasive method for constraining the likely neural locus of any resulting changes in auditory perception.

## Data Availability

Anonymised data are available on request from the authors.

## References

[1] Moore BCJ, Glasberg BR, Peters RW (1985). Relative dominance of individual partials in determining the pitch of complex tones. J Acoust Soc Am.

[2] Plomp R (1967). Pitch of complex tones. J Acoust Soc Am.

[3] Hoekstra A (1979) Frequency discrimination and frequency analysis in hearing. In: Institute of Audiology, University Hospital, Groningen, Netherlands.

[4] Houtsma AJM, Smurzynski J (1990). J.F.Schouten revisited: pitch of complex tones having many high-order harmonics. J Acoust Soc Am.

[5] Shackleton TM, Carlyon RP (1994). The role of resolved and unresolved harmonics in pitch perception and frequency modulation discrimination. J Acoust Soc Am.

[6] Moore BCJ, Carlyon RP, Plack CJ, Oxenham AJ (2005). Perception of pitch by people with cochlear hearing loss and by cochlear implant users. Springer Handbook of Auditory Research: Pitch Perception.

[7] Wouters J, McDermott HJ, Francart T (2015) Sound coding in cochlear implants. IEEE Signal Processing Magazine 32:67

[8] Coffey EBJ, Herholz SC, Chepesiuk AMP, Baillet S, Zatorre RJ (2016) Cortical contributions to the auditory frequency-following response revealed by MEG. Nat Commun 7 10.1038/ncomms11070 PMC4820836 27009409

[9] Coffey EBJ, Nicol T, White-Schwoch T, Chandrasekaran B, Krizman J, Skoe E, Zatorre RJ, Kraus N (2019) Evolving perspectives on the sources of the frequency-following response. Nat Commun 10 10.1038/s41467-019-13003-w PMC6834633 31695046

[10] Gorina-Careta N, Kurkela JLO, Hämäläinen J, Astikainen P, Escera C (2021) Neural generators of the frequency-following response elicited to stimuli of low and high frequency: a magnetoencephalographic (MEG) study. Neuroimage 231. 10.1016/j.neuroimage.2021.117866 33592244

[11] Holmes E, Purcell DW, Carlyon RP, Gockel HE, Johnsrude IS (2018). Attentional modulation of envelope-following responses at lower (93–109 Hz) but not higher (217–233 Hz) modulation rates. J Assoc Otolaryngol.

[12] Tichko P, Skoe E (2017). Frequency-dependent fine structure in the frequency-following response: the byproduct of multiple generators. Hear Res.

[13] Krishnan A, Plack CJ (2011). Neural encoding in the human brainstem relevant to the pitch of complex tones. Hear Res.

[14] Oxenham AJ, Keebler MV (2008) Complex pitch perception above the “existence region” of pitch. In: J Assoc Otolaryngol Midwinter Metting, p 140. Phoenix, AZ, USA

[15] Brown CJ, Etler C, He S, O’Brien S, Erenberg S, Kim JR, Dhuldhoya AN, Abbas PJ (2008). The electrically evoked auditory change complex: preliminary results from Nucleus cochlear implant users. Ear Hearing.

[16] He S, Grose JH, Teagle HFB, Buchman CA (2014). Objective measures of electrode discrimination with electrically evoked auditory change complex and speech-perception abilities in children with auditory neuropathy spectrum disorder. Ear Hearing.

[17] Martin BA, Boothroyd A (2000). Cortical, auditory, evoked potentials in response to changes of spectrum and amplitude. J Acoust Soc Am.

[18] Mathew R, Undurraga J, Li GP, Meerton L, Boyle P, Shaida A, Selvadurai D, Jiang D, Vickers D (2017). Objective assessment of electrode discrimination with the auditory change complex in adult cochlear implant users. Hear Res.

[19] Ostroff JM, Martin BA, Boothroyd A (1998). Cortical evoked response to acoustic change within a syllable. Ear Hearing.

[20] Presacco A, Middlebrooks JC (2018). Tone-evoked acoustic change complex (ACC) recorded in a sedated animal model. Jaro-Journal of the Association for Research in Otolaryngology.

[21] Gransier R, Carlyon RP, Wouters J (2020). Electrophysiological assessment of temporal envelope processing in cochlear implant users. Sci Rep.

[22] Undurraga JA, Van Yper L, Bance M, McAlpine D, Vickers D (2021). Neural encoding of spectro-temporal cues at slow and near speech-rate in cochlear implant users. Hear Res.

[23] Gransier R, Guérit F, Carlyon RP, Wouters J (2021). Frequency following responses and rate change complexes in cochlear implant users. Hear Res.

[24] Bernstein JG, Oxenham AJ (2003). Pitch discrimination of diotic and dichotic tone complexes: harmonic resolvability or harmonic number?. J Acoust Soc Am.

[25] Carlyon RP (1996). Masker asynchrony impairs the fundamental-frequency discrimination of unresolved harmonics. J Acoust Soc Am.

[26] Carlyon RP, Deeks JM (2002). Limitations on rate discrimination. J Acoust Soc Am.

[27] Richardson M, Guerit F, Harland A, Gransier R, Wouters J, Carlyon RP, Middlebrooks JC (2022). Temporal pitch sensitivity in an animal model: 1 Psychophysics and scalp recordings. J Assoc Res Otolaryngol Online first.

[28] Macherey O, Carlyon RP (2014). Re-examining the upper limit of temporal pitch. J Acoust Soc Am.

[29] Deeks JM, Gockel HE, Carlyon RP (2013). Further investigations of complex pitch perception in the absence of a place-rate match. J Acoust Soc Am.

[30] Carlyon RP, Guérit F, Deeks JM, Harland A, Gransier R, Wouters J, de Rijk SR, Bance ML (2021) Using interleaved stimulation to measure the size and selectivity of the sustained phase-locked neural response to cochlear-implant stimulation. J Assoc Res Otolaryngol 22:141-159. https://link.springer.com/article/10.1007/s10162-020-00783-y. 10.1007/s10162-020-00783-y PMC7943679 33492562

[31] Gockel HE, Carlyon RP, Mehta A, Plack CJ (2011). The Frequency Following Response (FFR) may reflect pitch-bearing information but is not a direct representation of pitch. J Assoc Res Otolaryngol.

[32] Gockel HE, Krugliak A, Plack CJ, Carlyon RP (2015). Specificity of the human frequency following response for carrier and modulation frequency assessed using adaptation. Jaro-Journal of the Association for Research in Otolaryngology.

[33] Gockel HE, Farooq R, Muhammed L, Plack CJ, Carlyon RP (2012) Differences between psychoacoustic and frequency following response measures of distortion tone level and masking. J Acoust Soc Am 132:2524–2535 10.1121/1.4751541 PMC5777604 23039446

[34] Dobie RA, Wilson MJ (1996). A comparison of t test, F test, and coherence methods of detecting steady-state auditory-evoked potentials, distortion-product otoacoustic emissions, or other sinusoids. J Acoust Soc Am.

[35] Elberling C, Kristensen SG, Don M (2012). Auditory brainstem responses to chirps delivered by different insert earphones. J Acoust Soc Am.

[36] Martin GK, Lonsbury-Martin BL, Probst R, Coats AC (1988). Spontaneous otoacoustic emissions in a non-human primate. I. Basic features and relations to other emissions. HearRes.

[37] Deprez H, Gransier R, Hofmann M, van Wieringen A, Wouters J, Moonen M (2017). Characterization of cochlear implant artifacts in electrically evoked auditory steady-state responses. Biomed Signal Process Control.

[38] Krishnan A (1999). Human frequency-following responses to two-tone approximations of steady-state vowels. Audiol Neurootol.

[39] Krishnan A (2006) Frequency-following response. In: Auditory evoked potentials: basic principles and clinical applications (Burkard R, Don M, Eggermont J, eds), pp 313–333

[40] Skoe E, Kraus N (2010). Auditory brain stem response to complex sounds: a tutorial. Ear Hearing.

[41] Elberling C, Don M, Cebulla M, Sturzebecher E (2007). Auditory steady-state responses to chirp stimuli based on cochlear traveling wave delay. J Acoust Soc Am.

[42] Ruggero MA, Temchin AN (2007). Similarity of traveling-wave delays in the hearing organs of humans and other tetrapods. Jaro-Journal of the Association for Research in Otolaryngology.

[43] Moore BCJ, Glasberg BR (1990). Frequency discrimination of complex tones with overlapping and non-overlapping harmonics. J Acoust Soc Am.

[44] Plack CJ, Carlyon RP (1995). Differences in frequency modulation detection and fundamental frequency discrimination between complex tones consisting of resolved and unresolved harmonics. J Acoust Soc Am.

[45] Plack CJ, Watkinson RK (2010). Perceived continuity and pitch shifts for complex tones with unresolved harmonics. J Acoust Soc Am.

[46] Mathias SR, Micheyl C, Bailey PJ (2010). Stimulus uncertainty and insensitivity to pitch-change direction. J Acoust Soc Am.

[47] Arzounian D, de Kerangal M, de Cheveigne A (2017). Sequential dependencies in pitch judgments. J Acoust Soc Am.

[48] Krumbholz K, Patterson RD, Pressnitzer D (2000). The lower limit of pitch as determined by rate discrimination. J Acoust Soc Am.

[49] Stahl P, Macherey O, Meunier S, Roman S (2016). Rate discrimination at low pulse rates in normal-hearing and cochlear implant listeners: influence of intracochlear stimulation site. J Acoust Soc Am.

[50] Bernstein JGW, Oxenham AJ (2006). The relationship between frequency selectivity and pitch discrimination: effects of stimulus level. J Acoust Soc Am.

[51] Bland JM, Altman DG (1995). Calculating correlation coefficients with repeated observations: part 1-correlation within subjects. BMJ.

[52] Don M, Kwong B, Tanaka C (2005). A diagnostic test for Meniere’s disease and cochlear hydrops: impaired high-pass noise masking of auditory brainstem response. Otol Neurotol.

[53] He S, Grose JH, Buchman CA (2012). Auditory discrimination: the relationship between psychophysical and electrophysiological measures. Int J Audiol.

[54] Martin BA, Boothroyd A (1999). Cortical, auditory, event-related potentials in response to periodic and aperiodic stimuli with the same spectral envelope. Ear Hearing.

[55] Carcagno S, Plack CJ (2011). Subcortical plasticity following perceptual learning in a pitch discrimination task. J Assoc Res Otolaryngol.

[56] Han JH, Dimitrijevic A (2015) Acoustic change responses to amplitude modulation: a novel method to quantify cortical temporal processing and hemispheric asymmetry. Front Neurosci 9 10.3389/fnins.2015.00038 PMC4324071 25717291

[57] Ritter S, Dosch HG, Specht HJ, Rupp A (2005). Neuromagnetic responses reflect the temporal pitch change of regular interval sounds. Neuroimage.

[58] Rupp A, Hauck M, Dosch HG, Patterson RD (2018). The effect of age on Huggins’ pitch processing and its location in auditory cortex. Acta Acust Acust.

[59] Seither-Preisler A, Patterson RD, Krumbholz K, Seither S, Luetkenhoener B (2006). From noise to pitch: transient and sustained responses of the auditory evoked field. Hear Res.

[60] Yost WA (1996). Pitch strength of iterated rippled noise. J Acoust Soc Am.

[61] Gockel H, Moore BCJ, Patterson RD, Meddis R (2003). Louder sounds can produce less forward, masking: effects of component phase in complex tones. J Acoust Soc Am.

[62] Kim JR, Brown CJ, Abbas PJ, Etler CP, O’Brien S (2009). The effect of changes in stimulus level on electrically evoked cortical auditory potentials. Ear Hear.

[63] Ortiz JA, Wright BA (2010). Differential rates of consolidation of conceptual and stimulus learning following training on an auditory skill. Exp Brain Res.

[64] Arzounian D, de Kerangal M, de Cheveigne A (2017). A sliding two-alternative forced-choice paradigm for pitch discrimination. J Acoust Soc Am.

[65] Jesteadt W, Bilger RC (1974). Intensity and frequency discrimination in one- and two-interval paradigms. The Journal of the Acoustical Society of America.

[66] Mathias SR, Bailey PJ, Semal C, Demany L (2011) A note about insensitivity to pitch-change direction. J Acoust Soc Am 130:EL129-EL134 10.1121/1.3629139 21974481

[67] Semal C, Demany L (2006). Individual differences in the sensitivity to pitch direction. J Acoust Soc Am.

[68] Carlyon RP, Deeks JM, Guérit F, Lamping W, Billig AJ, Large CH, Harris P (2018). Evaluation of possible effects of a potassium channel modulator on temporal processing by cochlear implant listeners. J Asssoc Res Otalryngol.

[69] Chambers AR, Pilati N, Balaram P, Large CH, Kaczmarek LK, Polley DB (2017) Pharmacological modulation of Kv3.1 mitigates auditory midbrain temporal processing deficits following auditory nerve damage. Sci Rep 7:17496 10.1038/s41598-017-17406-x PMC5727503 29235497

[70] Dieter A, Keppeler D, Moser T (2020). Towards the optical cochlear implant: optogenetic approaches for hearing restoration. EMBO Mol Med.

[71] Middlebrooks J, Snyder R (2009) Enhanced transmission of temporal fine structure using penetrating auditory nerve electrodes. In: Association for Research in Otolaryngology, 32nd Midwinter Research Meeting p328. Baltimore, Maryland, USA

[72] Vollmer M, Beitel RE, Schreiner CE, Leake PA (2017). Passive stimulation and behavioral training differentially transform temporal processing in the inferior colliculus and primary auditory cortex. J Neurophysiol.

